# Participation of Behavioral Health Facilities in Medicare Accountable Care Organizations

**DOI:** 10.1001/jamahealthforum.2024.4022

**Published:** 2024-11-27

**Authors:** Yucheng Hou, Susan H. Busch, Helen Newton

**Affiliations:** 1 Department of Management, Policy and Community Health, UTHealth Houston School of Public Health, Houston, Texas; 2 Department of Health Policy and Management, Yale School of Public Health, New Haven, Connecticut; 3 Department of Family Medicine, University of North Carolina School of Medicine, Chapel Hill

## Abstract

This cross-sectional study examines temporal trends in behavioral health facility inclusion in accountable care organizations (ACOs) and beneficiary characteristics of such ACOs.

## Introduction

Medicare and other payers are increasingly relying on value-based payment (VBP) models to provide coordinated high-quality care while containing costs. Although 1 in 3 Medicare beneficiaries has a behavioral health (BH) condition,^1^ integration of BH facilities has not been a focus of many Medicare VBP models. Accountable care organizations (ACOs) are networks of medical groups, hospitals, or other specialty care facilities that together contract to meet financial and quality benchmarks for an assigned patient population. ACOs are expected to cover all Medicare fee-for-service beneficiaries by 2030,^2^ including more than 1 million dual-eligible beneficiaries with complex BH needs and high spending.^3^ Community-based outpatient BH facilities provide most BH care for these beneficiaries and are increasingly offering primary care and other services, but whether these facilities are participating in ACO contracts over time is not known.

## Methods

In this repeated cross-sectional study, we linked several Medicare Shared Savings Program (MSSP) ACO Public Use Files and the National Plan & Provider Enumeration System to describe participation of BH facilities, including outpatient (community mental health centers and substance use disorder treatment programs) and institutional facilities (inpatient psychiatric facilities and units [IPFs]), in MSSP ACOs from 2014 to 2022 (eMethods and eTable in Supplement 1). We compared ACO and beneficiary characteristics among ACO-years with and without BH facility participants. We distinguished between ACO-years that included (1) any outpatient BH facilities, (2) any IPFs, (3) outpatient BH facilities only, and (4) IPFs only because of differences in participation mechanisms. This study followed the STROBE reporting guidelines and per the Common Rule was exempt from institutional review board review because it used publicly available data. Data were analyzed using Stata, version 18.0.

## Results

ACOs with at least 1 BH facility increased from 93 of 333 (28%) to 214 of 482 (44%) during 2014-2022, with a nearly constant average of 44% since 2019 (Figure). By 2022, 38% of ACOs included IPFs, compared with 19% of ACOs that included outpatient BH facilities. Only a small subset (6%) of ACOs included outpatient BH facilities only.

**Figure.**
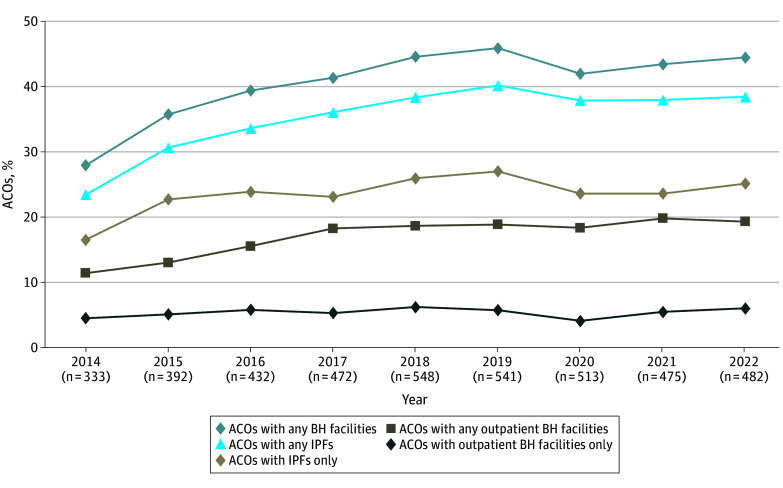
Percentage of Accountable Care Organizations (ACOs) With Behavioral Health (BH) Facilities Between 2014 and 2022 The total number of ACOs each year is given in parentheses. IPF indicates inpatient psychiatric facility.

Compared with ACO-years with no BH facilities, ACO-years with at least 1 BH facility had more attributed beneficiaries, were more likely to assume 2-sided (downside) risk, and had more safety-net providers in their network (Table). Among those with a BH facility, ACO-years with outpatient BH facilities, relative to ACO-years with IPFs, served a higher proportion of older dually eligible beneficiaries aged 65 years or older (9% vs 6%), those eligible for Medicare due to disability (15% vs 12%), or members of racial and ethnic minority groups (15% vs 13%).

**Table.** Summary Statistics of ACO-Years by BH Facility Participation (2014-2022)^a^

Characteristic	Mean (SD)					
	No BH facility	Any BH facility	Participation by BH facility type			
			Any outpatient BH facility^b^	Any IPF^c^	Outpatient BH facility only	IPF only
ACO-years, No. (%)	2386 (59)	1691 (41)	715 (18)	1470 (36)	221 (5)	976 (24)
ACO characteristics						
No. of assigned beneficiaries, thousands	13.73 (10.81)	28.02 (26.26)	32.50 (30.32)	29.79 (27.09)	16.30 (15.40)	24.74 (22.28)
Years in MSSP	2.84 (2.39)	2.98 (2.34)	3.10 (2.41)	2.91 (2.30)	3.40 (2.58)	2.88 (2.28)
2-Sided (downside) risks, No. (%)	490 (21)	389 (23)	209 (29)	333 (23)	56 (25)	180 (18)
Earned shared savings, No. (%)	1124 (47)	702 (42)	334 (47)	567 (39)	135 (61)	368 (38)
Had safety-net providers, No. (%)^d^	742 (31)	1098 (65)	537 (75)	940 (64)	158 (71)	561 (57)
Patient characteristics						
Average share of older dually eligible beneficiaries aged ≥65 y^e^	0.08 (0.10)	0.07 (0.07)	0.09 (0.08)	0.06 (0.05)	0.13 (0.11)	0.06 (0.05)
HCC risk score (dually eligible, aged ≥65 y)^f^	1.02 (0.12)	1.00 (0.09)	0.99 (0.10)	1.01 (0.08)	0.95 (0.13)	1.01 (0.09)
Average share of beneficiaries with disability^e^	0.12 (0.07)	0.13 (0.07)	0.15 (0.08)	0.12 (0.05)	0.20 (0.12)	0.12 (0.05)
HCC risk score (with disability)^f^	1.05 (0.16)	1.02 (0.11)	1.02 (0.12)	1.03 (0.11)	1.01 (0.10)	1.02 (0.11)
Average share of racial and ethnic minority beneficiaries^e^^,^^g^	0.17 (0.14)	0.14 (0.13)	0.15 (0.15)	0.13 (0.12)	0.21 (0.19)	0.14 (0.12)

Abbreviations: ACO, accountable care organization; BH, behavioral health; HCC, hierarchical chronic conditions; IPF, inpatient psychiatric facility; MSSP, Medicare Shared Savings Program.

^a^ Summary statistics of ACO-years (n = 4077) by BH facility participation (no BH facility, n = 2386; any BH facility, n = 1691) were derived by linking the identified BH facility participants to the ACO performance year financial and quality results public use files each year. ACO-years were excluded (3% of total ACO-years) if they had missing data for any of the variables listed in the first column.

^b^ Outpatient BH facilities may include residential beds.

^c^ IPFs include both inpatient psychiatric units in acute care hospitals and freestanding psychiatric hospitals.

^d^ Safety-net providers included federally qualified health centers, rural health clinics, and critical access hospital participants.

^e^ Share range, 0-1. Calculated as the number of assigned beneficiaries who were dually eligible for Medicaid (aged ≥65 y), had disabilities, or were members of racial and ethnic minority groups divided by the total number of assigned beneficiaries each ACO-year.

^f^ Average HCC risk scores by enrollment types (older dual, disability) for ACO baseline year 3.

^g^ The racial and ethnic minority group included beneficiaries who are American Indian or Alaska Native, Asian and Pacific Islander, Black, Hispanic, other, and unknown; we analyzed racial and ethnic minority beneficiaries collectively, given the Centers for Medicare & Medicaid Services data suppression rule for services delivered to 10 or fewer beneficiaries.

## Discussion

The proportion of ACOs with BH facilities was increasing before 2019 but still remained low overall (44%). The trend leveled off as the number of ACOs, especially those serving beneficiaries with high medical complexity,^4^ decreased following MSSP rule changes and the COVID-19 pandemic.

That fewer than 1 in 5 ACOs (19%) contracted with an outpatient BH facility is concerning. Including outpatient BH facilities in ACO contracts could be instrumental in coordinating BH and physical care for attributed beneficiaries, especially for ACOs that serve a higher proportion of underserved and rural populations, as outpatient BH facilities may be the only source of specialty BH services in the community. Our findings reflect the challenges of integrating outpatient BH facilities into VBP models, including ACOs’ pressure to bear downside risk, lack of BH-specific quality metrics, or limited experience with risk-based contracts among outpatient BH facilities.^3,5^ As the Centers for Medicare & Medicaid Services continues to seek alternative payment models to improve BH treatment access,^2^ including the newly introduced Innovation in Behavioral Health model,^6^ understanding current patterns of outpatient BH facility participation in VBP models can help establish adequate participant network standards for future models.^5^

Limitations include that the data may not identify all BH facilities participating in ACOs and do not capture informal relationships between BH facilities and ACOs. Identifying BH facility participants may exclude clinicians with BH specialties practicing in other settings.

## Notes

Supplement 1. **eMethods.** Identifying Behavioral Health (BH) Facilities Participating in the Medicare Shared Savings Program (MSSP) Accountable Care Organizations (ACOs) **eTable.** Number of ACOs With Behavioral Health Facility Participants, 2014-2022

Supplement 2.
Data Sharing Statement
