# Error in Funding/Support in Article Information

**DOI:** 10.1001/jamahealthforum.2024.5462

**Published:** 2025-01-31

In the Research Letter titled “Participation of Behavioral Health Facilities in Medicare Accountable Care Organizations,”^1^ published November 27, 2024, there was an error in the Funding/Support section of Article Information. The grant number given in Funding/Support as R01DA057789 should have been R01DA049757. In addition, the Support noted did contribute to this work, so the phrase “outside the submitted work” has been removed from the Funding/Support acknowledgement. This article has been corrected.
