# Supplementary material for: Participation of Behavioral Health Facilities in Medicare Accountable Care Organizations
Source: JAMA Health Forum. 2024 Nov 27;5(11):e244022. doi: 10.1001/jamahealthforum.2024.4022 (PMC11786226; doi:10.1001/jamahealthforum.2024.4022)
Supplement: Supplement 1. — eMethods. Identifying Behavioral Health (BH) Facilities Participating in the Medicare Shared Savings Program (MSSP) Accountable Care Organizations (ACOs) eTable. Number of ACOs With Behavioral Health Facility Participants, 2014-2022 [file jamahealthforum-e244022-s001.pdf]

Supplemental Online Content

Hou Y, Busch SH, Newton H. Participation of behavioral health facilities in Medicare accountable care organizations. *JAMA Health Forum*. 2024;5(11):e244022. doi:10.1001/jamahealthforum.2024.4022

eMethods. Identifying Behavioral Health (BH) Facilities Participating in the Medicare Shared Savings Program (MSSP) Accountable Care Organizations (ACOs)

eTable. Number of ACOs With Behavioral Health Facility Participants, 2014-2022

This supplemental material has been provided by the authors to give readers additional information about their work.

© 2024 Hou Y et al. *JAMA Health Forum.*

eMethods.

Identifying Behavioral Health (BH) Facilities Participating in the Medicare Shared Savings Program (MSSP) Accountable Care Organizations (ACOs)

We identified BH facilities participating in MSSP ACOs in the following steps. First, we linked the unique legal business name (primary match) associated with the ACO participant's federal tax identification number from the ACO public use files each year (2014-2022) to the organizational providers registered in the National Plan & Provider Enumeration System (NPPES) (May 2023 version). The primary match was supplemented by considering other available names recorded in NPPES as secondary identifiers, including other organization names and parent organization names. Second, the ACO participants matched in NPPES were verified by the state location recorded in NPPES and ACO service areas. The first two steps resulted in an average matching rate of 80% across all years; the matching rate after each step is documented below. Third, we identified different types of BH facilities using the organizational taxonomy codes for BH facilities eligible for Medicare programs selected from the Medicare Provider and Supplier Taxonomy Crosswalk (September 2023 version). Medicares-eligible taxonomy codes for outpatient BH facilities include mental health clinic/center (including community mental health center, 261QM0801X) and substance use disorder rehabilitation clinic/center (261QR0405X). Medicare-eligible taxonomy codes for institutional BH facilities include hospital psychiatric unit (273R00000X), hospital rehabilitation/substance use disorder unit (276400000X), and psychiatric hospital (283Q00000X).

ACO Participants Matched to National Plan & Provider Enumeration System, 2014-2022

	**2014**	**2015**	**2016**	**2017**	**2018**	**2019 (Jan)**	**2019 (July)**	**2020**	**2021**	**2022**
No. of unique ACO participants	13648	15185	14463	17032	19273	17861	17805	17023	15983	15705
No. (%) of ACO participants matched to NPPES (primary)	9114 (67)	10876 (72)	11275 (78)	13381 (79)	15516 (81)	14680 (82)	14881 (84)	14828 (87)	14017 (88)	14067 (90)
No. (%) of ACO participants matched to NPPES (primary+secondary)	9248 (68)	11022 (73)	11382 (79)	13496 (79)	15639 (81)	14779 (83)	14978 (84)	14917 (88)	14101 (88)	14137 (90)
No. (%) of ACO participants matched to NPPES (primary+secondary+state location)	8988 (66)	10826 (71)	11249 (78)	13317 (78)	15454 (80)	14502 (81)	14721 (83)	14709 (86)	13864 (87)	13843 (88)

© 2024 Hou Y et al. *JAMA Health Forum.*

eTable. Number of ACOs with Behavioral Health Facility Participants, 2014-2022

	2014	2015	2016	2017	2018	2019[a]	2020	2021	2022
Total No. of ACOs[b]	333	392	432	472	548	541[c]	513	475	482
No. (%) of ACOs with any BH facilities	93 (28)	140 (36)	170 (39)	195 (41)	244 (45)	248 (46)	215 (42)	206 (43)	214 (44)
No. (%) of ACOs with any outpatient BH facilities[d]	38 (11)	51 (13)	67 (16)	86 (18)	102 (19)	102 (19)	94 (18)	94 (20)	93 (19)
No. (%) of ACOs with any psychiatric hospitals/units[d]	78 (23)	120 (31)	145 (34)	170 (36)	210 (38)	217 (40)	194 (38)	180 (38)	185 (38)
No. (%) of ACOs with only outpatient BH facilities	15 (5)	20 (5)	25 (6)	25 (5)	34 (6)	31 (6)	21 (4)	26 (5)	29 (6)
No. (%) of ACOs with only psychiatric hospitals/units	55 (17)	89 (23)	103 (24)	109 (23)	142 (26)	146 (27)	121 (24)	112 (24)	121 (25)

Abbreviations: ACO, accountable care organization; BH, behavioral health.

Notes: [a]2019 contains one 12-month performance period and two six-month performance periods to facilitate existing and new ACOs' transition to "Pathways to Success" beginning on July 1, 2019.

[b]Number of ACOs reported financial and quality performance in the ACO public use files.

[c]Included 309 ACOs participated in the full 12-month performance period, 139 ACOs participated in the first 6-month performance period (Jan 1, 2019-June 30, 2019) and entered a new participation agreement in the second 6-month performance period (July 1, 2019-Dec 31, 2019), 27 ACOs participated in the first 6-month performance period and exited the program, and 66 new ACOs started in the second 6-month performance period.

[d]The numbers do not sum to the total number of ACOs with any BH facilities because ACOs can have both outpatient and institutional BH participants.

© 2024 Hou Y et al. *JAMA Health Forum.*
