# Supplementary material for: Participation of Behavioral Health Facilities in Medicare Accountable Care Organizations
Source: JAMA Health Forum. 2024 Nov 27;5(11):e244022. doi: 10.1001/jamahealthforum.2024.4022 (PMC11786226; doi:10.1001/jamahealthforum.2024.4022)
Supplement: Supplement 2. — Data Sharing Statement [file jamahealthforum-e244022-s002.pdf]

Data Sharing Statement

Hou. Participation of Behavioral Health Facilities in Medicare Accountable Care Organizations. *JAMA Health Forum*. Published November 27, 2024. doi:10.1001/jamahealthforum.2024.4022

Data
Data available: No

Additional Information
Explanation for why data not available: All data used in this study are publicly available. Data on accountable care organizations are available via https://www.cms.gov/medicare/payment/fee-for-service-providers/shared-savings-program-ssp-acos/data. Processed versions of National Plan and Provider Enumeration System (NPPES) are available via National Bureau of Economic Research public use files (https://www.nber.org/research/data/national-plan-and-provider-enumeration-system-nppes).
